# Development of a Subtraction Processing Technology for Assistance in the Comparative Interpretation of Mammograms

**DOI:** 10.3390/diagnostics14111131

**Published:** 2024-05-29

**Authors:** Chiharu Kai, Satoshi Kondo, Tsunehiro Otsuka, Akifumi Yoshida, Ikumi Sato, Hitoshi Futamura, Naoki Kodama, Satoshi Kasai

**Affiliations:** 1 Department of Radiological Technology, Faculty of Medical Technology, Niigata University of Health and Welfare, Niigata City 950-3198, Japan; chiharu-kai@nuhw.ac.jp (C.K.); akifumi-yoshida@nuhw.ac.jp (A.Y.); kodama@nuhw.ac.jp (N.K.); 2 Major in Health and Welfare, Graduate School of Niigata University of Health and Welfare, Niigata City 950-3198, Japan; ikumi-sato@nuhw.ac.jp; 3 Graduate School of Engineering, Muroran Institute of Technology, Muroran City 050-8585, Japan; 4 Otsuka Breastcare Clinic, Tokyo 121-0813, Japan; 5 Department of Nursing, Faculty of Nursing, Niigata University of Health and Welfare, Niigata City 950-3198, Japan; 6 Konica Minolta, Inc., Tokyo 100-7015, Japan

**Keywords:** mammogram, registration, subtraction, breast cancer, comparative interpretation

## Abstract

A comparative interpretation of mammograms has become increasingly important, and it is crucial to develop subtraction processing and registration methods for mammograms. However, nonrigid image registration has seldom been applied to subjects constructed with soft tissue only, such as mammograms. We examined whether subtraction processing for the comparative interpretation of mammograms can be performed using nonrigid image registration. As a preliminary study, we evaluated the results of subtraction processing by applying nonrigid image registration to normal mammograms, assuming a comparative interpretation between the left and right breasts. Mediolateral-oblique-view mammograms were taken from noncancer patients and divided into 1000 cases for training, 100 cases for validation, and 500 cases for testing. Nonrigid image registration was applied to align the horizontally flipped left-breast mammogram with the right one. We compared the sum of absolute differences (SAD) of the difference of bilateral images (Difference Image) with and without the application of nonrigid image registration. Statistically, the average SAD was significantly lower with the application of nonrigid image registration than without it (without: 0.0692; with: 0.0549 (*p* < 0.001)). In four subgroups using the breast area, breast density, compressed breast thickness, and Difference Image without nonrigid image registration, the average SAD of the Difference Image was also significantly lower with nonrigid image registration than without it (*p* < 0.001). Nonrigid image registration was found to be sufficiently useful in aligning bilateral mammograms, and it is expected to be an important tool in the development of a support system for the comparative interpretation of mammograms.

## 1. Introduction

Mammography is the first modality of choice for breast cancer screening. Mammography screening has been proven to reduce breast cancer mortality [1,2,3]. Continuous mammography screening is also effective in reducing the mortality rate [4].

Breast radiologists assess the morphological characteristics of single-view mammograms to detect and diagnose benign and malignant breast cancers. With the development of digital technology, reading assistance tools for mammograms based on artificial intelligence (AI) have been developed and commercialized [5]. Examples include “CADe”, which supports detection, and “CADx”, which supports discrimination, as well as “CADe + CADx”, which supports both detection and discrimination. “CADt” has also been commercialized, presenting breast radiologists with the signs of an emergency. These assistance tools have been used clinically, and some reports have evaluated their usefulness [6,7,8,9,10,11]. Breast radiologists also compare bilateral and temporal mammograms as references to assess the differences in findings [12]. This comparative interpretation is important for the diagnosis of breast cancer, because an overlap of mammary tissues can make it difficult to distinguish between normal and abnormal tissues. However, few AI-based assistance tools support a comparative interpretation using multiple mammograms because of the difficulty in aligning them. Nevertheless, a tool that can subtract chest X-ray images captured at different times has been commercialized [13]. The use of this tool resulted in a superior performance for all observers in diagnosing lung cancer on chest radiographs (the mean Az value increased from 0.764 to 0.836, *p* = 0.0006). This tool can assist in the detection of abnormal chest findings by highlighting newly appearing abnormal findings or changes in the original findings. If this subtraction processing technology can be applied to mammograms to highlight areas of change, it could objectively assist with comparative interpretation. The importance of a comparative interpretation of mammograms is increasing, and it is important to develop subtraction processing technologies for mammograms.

Image registration is important for subtraction processing. The chest is covered with hard tissue, represented by the ribs, and is relatively easy to align during subtraction processing. However, it is difficult to align mammograms because the breast is made of soft tissue. For example, the positioning skill of radiographers tends to vary, and bilateral mammograms are not perfectly symmetrical, although the mammary glands tend to be of equal volume on both sides [14]. The VoxelMorph technique reported by Balakrishnan et al. [15] is a registration method for nonrigid objects such as body parts and organs. VoxelMorph is a framework that rapidly learns the best registration model for an entire dataset; however, the dataset is not large. In the original paper [15] and other research work [16,17,18,19], although the registration of MRI and CT scans was studied using VoxelMorph, there was no report on its application to subjects constructed with soft tissue only, such as mammograms.

Hence, the purpose of this study was to examine whether a subtraction processing technology for the comparative interpretation of mammograms can be constructed using VoxelMorph and applied to nonrigid mammograms. As a preliminary study, we evaluated the results of the subtraction processing technology by applying VoxelMorph to the mammograms of normal cases, assuming a comparative interpretation between the left and right breasts.

## 2. Materials and Methods

This study was approved by the Institutional Review Board of Niigata University of Health and Welfare (Approval No. 19221-240124). The details of the methods used to conduct the experiments are described in the following sections.

### 2.1. Data

We used mediolateral-oblique-view mammograms (Pe-ru-ru; CANON MEDICAL SYSTEMS CORPORATION, Tochigi, Japan). The mammograms were collected from the Otsuka Breastcare Clinic between March 2021 and March 2022. A total of 1600 normal cases was randomly selected, excluding cases with cancer, postoperative findings, or coarse calcifications. We initially evaluated normal cases to determine the effectiveness of our subtraction method. We randomly divided 1000, 100, and 500 cases for training, validation, and testing, respectively. The mammograms used in this study were collected by KONICA MINOLTA, INC., and shared as anonymously processed information. The company played no role in the study design, analysis, model development, or manuscript preparation.

### 2.2. Registration Method and Subtraction Processing

**Figure 1:**
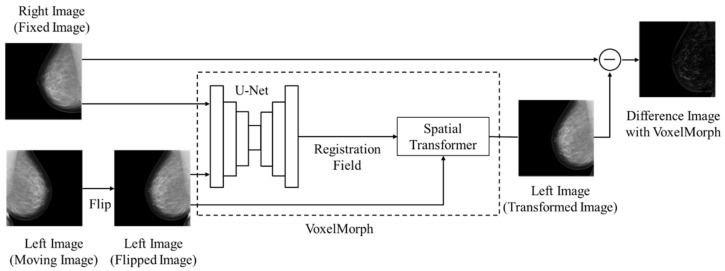
Architecture of the subtraction processing technology using VoxelMorph.

Figure 1 shows the overall architecture of the subtraction processing technology used in this study. VoxelMorph can learn to capture the exact location of mammogram features using U-Net [20]. The right-breast mammogram was treated as the reference image for registration (hereinafter “Fixed Image”) and the left-breast mammogram was treated as the moving image for registration (hereinafter “Moving Image”). After the horizontal flipping of the Moving Image (hereinafter “Flipped Image”), the Fixed Image and Flipped Image were inputted to VoxelMorph. First, to transform the Flipped Image to fit the Fixed Image, a deformation field from the Flipped Image to the Fixed Image was computed using U-Net. We used the same U-Net structure as in the original study [15]. Based on the obtained deformation field, the Flipped Image was transformed using a Spatial Transformer [21] (hereinafter, the “Transformed Image”). The absolute Difference Image between the Fixed Image and Transformed Image (hereinafter “Difference Image with VoxelMorph”) was finally outputted.

The mammograms used in this study were taken with an image size of 2016 × 2816 (pixel pitch: 85 µm). During preprocessing, padding was applied to the nipple side while maintaining the aspect ratio, and the image was resized to 520 × 520 (pixel pitch: 200 μm) using bicubic interpolation (4 × 4 neighborhood). Subsequently, the center of the image was cropped to obtain an image with a size of 512 × 512 pixels. As in the original study [15], the loss function is defined according to Equation (1).

(1) $$L\left(f,m,\emptyset \right)=L_{mse}\left(f,m\circ \emptyset \right)+\lambda L_{ppm}(\emptyset ),$$

where f, m, and ∅ are the Fixed Image, the Transformed Image, and a displacement field, respectively. $L_{mse}$ is the sum of the mean-squared error between the Fixed and the Transformed Images and $L_{ppm}$ is the per-pixel misalignment. The per-pixel misalignment is the mean of the squared spatial gradients of displacement for all pixels. It was learned so that the weighted sum of the mean-square error and the sum of the per-pixel misalignments would be low. The loss function was calculated only in the region of the breast and pectoralis major muscle extracted by means of skin line detection using raw data before image processing. The number of epochs was 800, the batch size was 32, *λ* was 1, the initial learning rate was 2 × 10^−4^, the optimization function was Adam, and the learning rate was varied for each epoch using cosine annealing. The average of the sum of absolute differences (hereinafter “SAD”) between the Fixed Image and Transformed Image was used as the objective index. We used a computer with a Ryzen 7 5800X CPU, 64 GB of main memory, and an NVIDIA GeForce RTX 3090 GPU for processing, and Python (version 3.9.5; Python Software Foundation, Wilmington, DE, USA) on PyTorch framework (version 2.0.1).

### 2.3. Evaluation Method

To confirm the applicability of the subtraction processing technology with VoxelMorph to the mammograms of normal cases, we compared the SAD of the absolute Difference Image between Fixed and Flipped Images before registration (Difference Image without VoxelMorph) and the Difference Image with VoxelMorph. We also assessed the improvement in the SAD using VoxelMorph. In addition to evaluating 500 cases for testing, we assessed factors that might affect the registration of the left- and right-breast mammograms. We used breast area, the mammary gland content ratio as breast density, compressed breast thickness, and Difference Image without VoxelMorph. To evaluate the mammograms by breast size, we divided the test cases into four groups of 125 cases each (breast area: Groups 1–4) in decreasing order of the percentage of breast area to the entire image. To evaluate the mammograms by mammary tissue volume, we divided the test cases into four groups of 125 cases each (mammary gland content ratio: Groups 1–4) in decreasing order of the outputted value by our developed AI for estimating the mammary gland content ratio [22], which could be quantitatively evaluated with a high correlation by an expert doctor who developed the guidelines. To evaluate the mammograms by breast volume, we divided the test cases into four groups of 125 cases each (compressed breast thickness: Group 1–4) in decreasing order of the thickness between the compression and detection plates. To evaluate the bilateral differences in the mammary tissue before registration, we divided the test cases into four groups of 125 cases each (Difference Image without VoxelMorph: Groups 1–4) in increasing order of the SAD of the Difference Image without VoxelMorph. Table 1 lists the mean values for each subgroup.

## 3. Results

**Figure 2:**
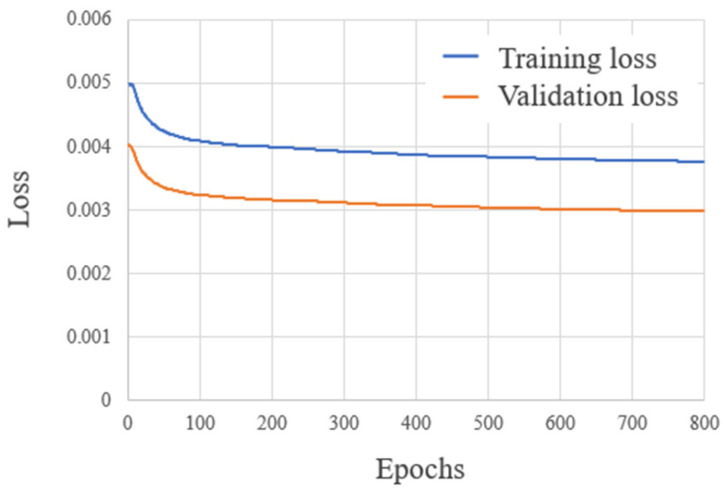
Learning curve for VoxelMorph.

Figure 2 shows the learning curve for training and validation, with the number of epochs (Epochs) on the horizontal axis and the loss value of Equation (1) (Loss) on the vertical axis. Training and validation losses were 0.00499 and 0.00403 and 0.00377 and 0.00299 at Epoch 1 and Epoch 800, respectively. Both training and validation losses improved as the number of the epochs progressed, but converged as they approached to 0.0037 and 0.0030, respectively.

**Figure 3:**
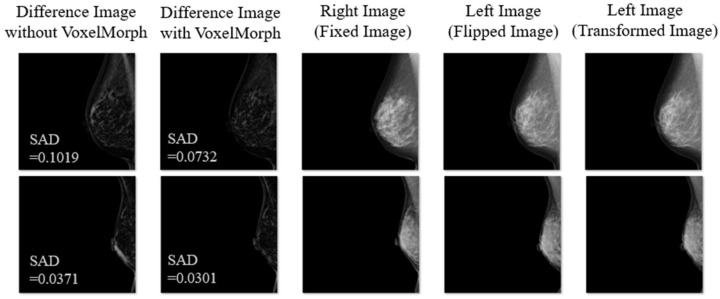
Representative examples of the experimental result (Difference Image without VoxelMorph, Difference Image with VoxelMorph, Fixed Image, Flipped Image, and Transformed Image).

Figure 3 shows the representative examples of the experimental results (Difference Images without VoxelMorph, Difference Images with VoxelMorph, and Fixed, Flipped, and Transformed Images). VoxelMorph corrected the deviation during the subtraction process near the edges, such as near the skin line of the breast and near the pectoralis muscle.

**Figure 4:**
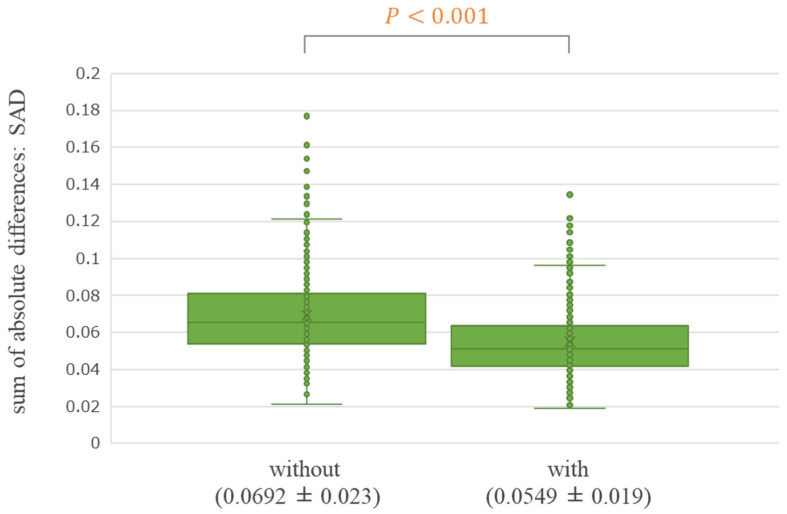
Comparison of the sum of absolute differences (SAD) between “Difference Image without VoxelMorph” and “Difference Image with VoxelMorph” (overall) (SAD average ± standard deviation).

Figure 4 shows a boxplot of the SAD of the Difference Image without VoxelMorph and Difference Image with VoxelMorph for the 500 test cases. Statistically, the average SAD of the Difference Image was significantly lower with VoxelMorph (SAD average ± standard deviation: 0.0549 ± 0.019) compared with that without VoxelMorph (SAD average ± standard deviation: 0.0692 ± 0.023) (*p* < 0.001). The variability was also reduced within the 500 cases. Table 2 summarizes the SAD results for the Difference Images without VoxelMorph and Difference Image with VoxelMorph for each subgroup. In all the subgroups, statistically, the average SAD of the Difference Image was significantly lower with VoxelMorph than without it (*p* < 0.001).

**Figure 5:**
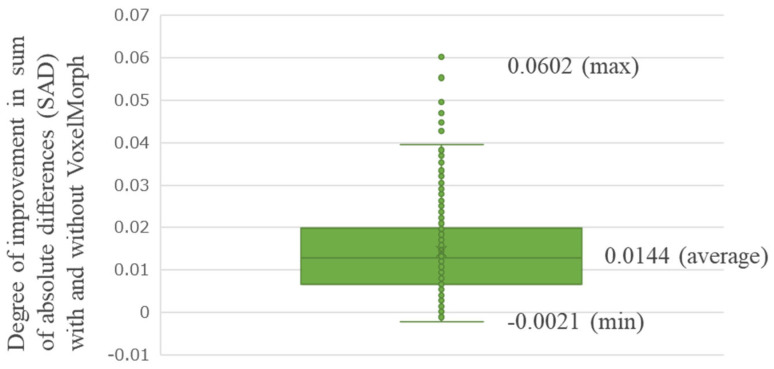
Comparison of the difference in the sum of absolute difference (SAD) averages between “Difference Image without VoxelMorph” and “Difference Image with VoxelMorph” (improvement in the SAD with and without VoxelMorph): overall.

**Figure 6:**
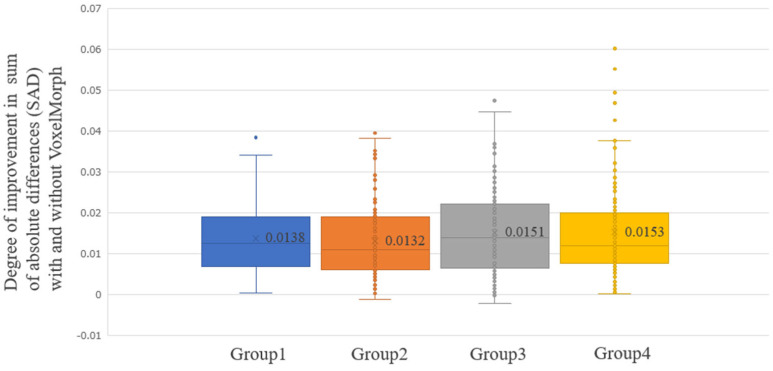
Comparison of the difference in the sum of absolute differences (SAD) between “Difference Image without VoxelMorph” and “Difference Image with VoxelMorph” (improvement in the SAD with and without VoxelMorph): Breast Area (%).

**Figure 7:**
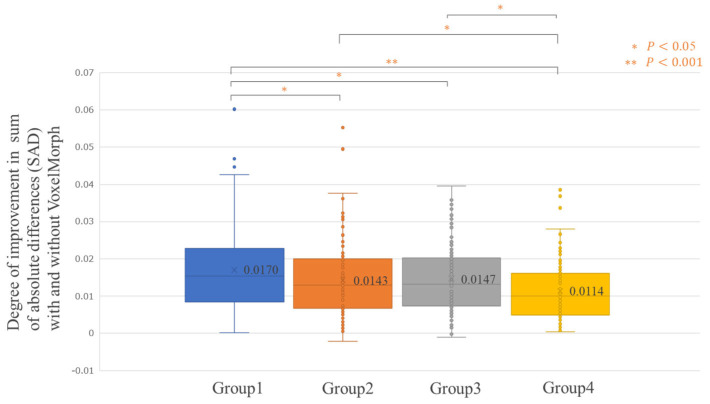
Comparison of the difference in the sum of absolute differences (SAD) between “Difference Image without VoxelMorph” and “Difference Image with VoxelMorph” (improvement in the SAD with and without VoxelMorph): mammary gland content ratio (%).

**Figure 8:**
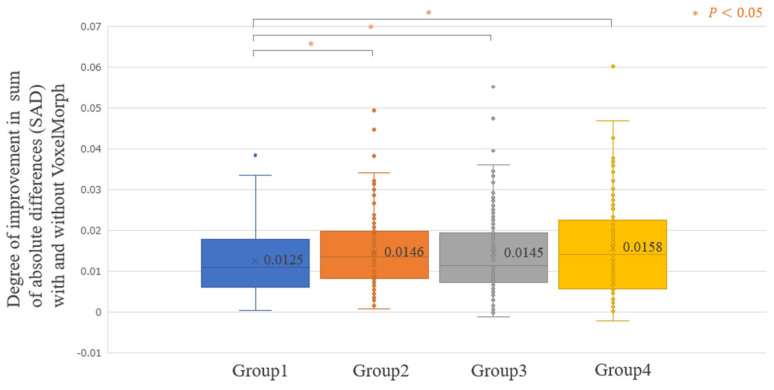
Comparison of the difference in the sum of absolute differences (SAD) between “Difference Image without VoxelMorph” and “Difference Image with VoxelMorph” (improvement in the SAD with and without VoxelMorph): compressed breast thickness (mm).

**Figure 9:**
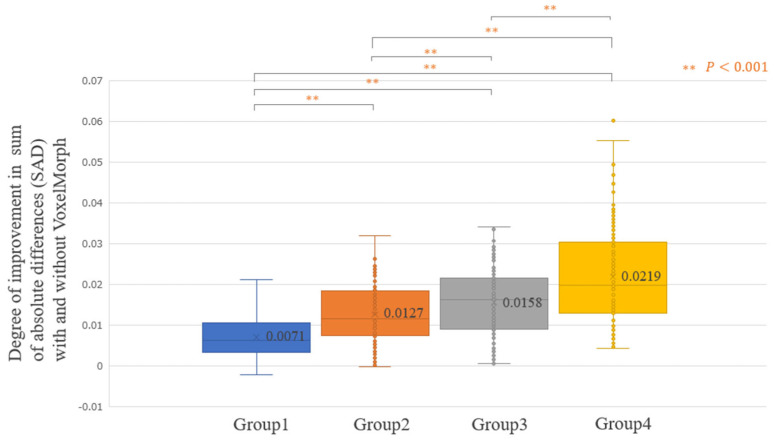
Comparison of the difference in the sum of absolute differences (SAD) between “Difference Image without VoxelMorph” and “Difference Image with VoxelMorph” (improvement in the SAD with and without VoxelMorph): Difference Image before VoxelMorph (SAD) application.

Figure 5 shows the average improvement in the SAD realized using VoxelMorph, evaluated for all the 500 testing cases (Improvement SAD average [min–max]: 0.0144 [−0.0021–0.0602]). For some patients, the SAD improved significantly; however, there were cases where the SAD did not improve. Figure 6, Figure 7, Figure 8 and Figure 9 show the improvement in the SAD realized using VoxelMorph, plotted for each subgroup. Figure 6 shows no statistically significant difference in the average improvement in the SAD between the breast area groups. Figure 7 shows that the average improvement in the SAD between the mammary gland content ratio groups was significantly higher (*p* < 0.05) in Group 1 than in Groups 2–4, in Group 2 than in Group 4, and in Group 3 than in Group 4. Figure 8 shows that the average improvement in the SAD between the compressed breast thickness groups was significantly higher (*p* < 0.05) in Group 4 than in Group 1, Group 3 than in Group 1, and Group 2 than in Group 1. Figure 9 shows that the average improvement in the SAD between the Difference Images without VoxelMorph groups was statistically significantly higher (*p* < 0.001) in Group 4 than in Groups 1–3, Group 3 than in Groups 2 and 3, and Group 2 than in Group 1.

## 4. Discussion

Our study results indicate that VoxelMorph is sufficiently effective in aligning the mammograms of the left and right breasts. This was observed in both the 500 cases for testing and the subgroup analyses. Although the soft tissue of the breast makes it difficult to align mammograms, VoxelMorph proved useful. This indicates that the development of a subtraction processing technology for mammograms is promising.

Statistically, the improvement in the SAD realized using VoxelMorph was significantly higher in the group with a lower mammary gland content ratio and a thicker compressed breast, particularly in the group with a higher SAD of the Difference Image without VoxelMorph. In cases with a low mammary gland content ratio and a thick compressed breast, the areas of the mammary tissue on the mammogram are often scattered and unlikely to be distributed in the same location between the right and left breasts. By applying VoxelMorph, we believe that the regional mammary distribution was successfully learned to fit between the right and left breasts, resulting in a higher degree of improvement in the SAD.

**Figure 10:**
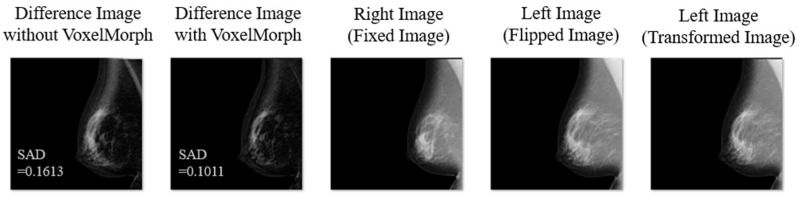
Subtraction processing results of mammograms in which the breast size and the spread of the mammary gland are very different between the left and right breasts.

**Figure 11:**
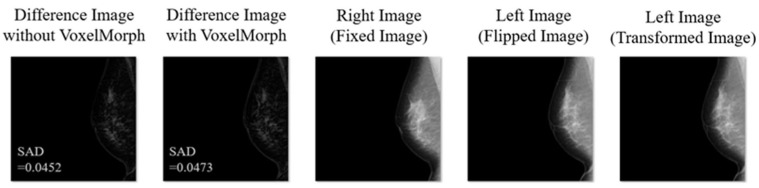
Subtraction processing results for mammograms in which the sum of absolute differences (SAD) does not improve with VoxelMorph.

Figure 10 shows the subtraction processing results of the mammograms, in which the breast size and the spread of the mammary gland are very different between the left and right breasts. A significant improvement in the SAD can be seen in response to the use of VoxelMorph. Therefore, VoxelMorph was found to be effective even when the breast shape differed significantly between the right and left sides. Figure 11 shows the subtraction processing results for mammograms in which the SAD did not improve with VoxelMorph, as shown in Figure 5. Such cases where the SAD did not improve were sufficiently small even without using VoxelMorph, with an incidence rate of 3 out of 500 cases (0.6%). Therefore, we believe that there are no negative effects in using VoxelMorph in the subtraction processing technology for mammograms.

**Figure 12:**
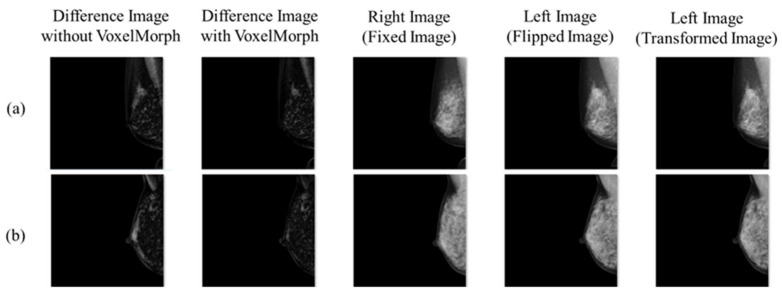
Results of applying the subtraction processing technology to two cancer cases: (**a**) a mass is detected in the upper region, and (**b**) calcification and focal asymmetric density (FAD) are detected in the upper region.

In this study, experiments were conducted on the mammograms of normal cases, assuming a comparative interpretation of the left and right breasts as a preliminary analysis. However, considering actual clinical utilization, it is necessary to consider the application of the subtraction processing technology with prior images as well as cancer cases. The subtraction processing technology with prior images could be applied in this experiment by replacing the Fixed Image with the prior image and the Flipped Image with the current image. However, regarding its application in cancer cases, it is necessary to compare the results of the subtraction processing in normal cases with those in cancer cases to determine the extent of deviation. Figure 12 shows the results of applying the subtraction processing technology used in this experiment to two cancer cases. Figure 12a shows a case in which a mass is detected in the upper region. Figure 12b shows a case in which calcification and focal asymmetric density (FAD) are detected in the upper region. In both cases, only areas with abnormal findings were highlighted in the subtraction-processed images using VoxelMorph, indicating a reading assistance effect. A limitation of this study is that this system has not been applied to many cancer cases to evaluate them in comparison with normal cases. Therefore, in the future, it will be necessary for physicians to conduct reading experiments using these subtraction-processed images and conduct research to evaluate the discrepancies between normal and cancer cases. We also have several limitations such as the limited case number, single- institution study, and no improvement on VoxelMorph network architecture. Therefore, it could be improved by conducting experiments on large data sets from multiple institutions, including the optimization of VoxelMorph, to clarify the clinical usefulness.

## 5. Conclusions

The results of the subtraction processing technology using VoxelMorph for the mammograms of normal cases were evaluated assuming a comparative interpretation between the left and right breasts. VoxelMorph was found to be sufficiently useful in aligning the left- and right-breast mammograms. It is expected to be an important tool in the development of a support system for the comparative interpretation of mammograms, which has become increasingly important in recent years.

## Figures and Tables

**Table 1:** Characteristic values for each of the subgroups (average ± standard deviation [min–max]).

		All	Group 1	Group 2	Group 3	Group 4
Breast area (%)	Training	33.3 ± 10.0	22.2 ± 2.9	29.0 ± 1.6	35.1 ± 2.0	47.0 ± 7.1
		[12.7–73.1]	[12.7–26.0]	[26.1–31.8]	[31.8–38.8]	[38.9–73.1]
	Validation	32.3 ± 8.6	22.5 ± 2.7	29.3 ± 2.1	34.4 ± 1.0	43.1 ± 7.6
		[14.9–64.2]	[14.9–25.7]	[25.9–32.4]	[32.6–36.2]	[36.2–64.2]
	Test	34.4 ± 10.7	22.8 ± 3.5	29.7 ± 1.4	36.0 ± 2.4	49.2 ± 7.4
		[11.1–79.8]	[11.1–27.1]	[27.2–32.4]	[32.4–40.7]	[40.8–79.8]
Mammary gland content ratio (%)	Training	45.0 ± 16.5	23.9 ± 6.2	38.5 ± 3.2	50.7 ± 4.4	66.7 ± 5.9
		[11.0–85.6]	[11.0–32.6]	[32.7–43.8]	[43.8–58.3]	[58.3–85.6]
	Validation	45.1 ± 15.1	26.5 ± 6.6	39.2 ± 3.0	49.4 ± 3.6	65.3 ± 5.9
		[11.8–81.0]	[11.8–33.8]	[34.0–43.5]	[43.6–56.9]	[57.7–81.0]
	Test	44.0 ± 16.0	24.0 ± 6.2	37.6 ± 3.0	49.0 ± 4.0	65.3 ± 6.9
		[11.5–85.0]	[11.5–32.7]	[32.8–42.5]	[42.5–56.5]	[56.6–85.0]
Compressed breast thickness (mm)	Training	44.7 ± 13.4	27.9 ± 4.7	39.6 ± 2.9	49.1 ± 2.9	62.2 ± 6.7
		[10–86]	[10–34]	[34–44]	[44–54]	[54–86]
	Validation	44.1 ± 12.4	29.0 ± 4.7	38.7 ± 2.8	48.7 ± 3.0	59.9 ± 6.4
		[20–78]	[20–34]	[34–44]	[44–54]	[54–78]
	Test	45.7 ± 13.6	27.9 ± 5.0	40.8 ± 3.2	51.0 ± 2.8	63.0 ± 5.6
		[12–84]	[12–36]	[36–46]	[46–56]	[56–84]
Difference Image without VoxelMorph (sum of absolute differences: SAD)	Training	0.070 ± 0.025	0.044 ± 0.006	0.058 ± 0.004	0.073 ± 0.005	0.105 ± 0.023
		[0.022–0.230]	[0.022–0.052]	[0.052–0.065]	[0.065–0.082]	[0.082–0.230]
	Validation	0.069 ± 0.023	0.044 ± 0.006	0.059 ± 0.004	0.072 ± 0.006	0.101 ± 0.014
		[0.031–0.128]	[0.031–0.051]	[0.052–0.063]	[0.064–0.083]	[0.084–0.128]
	Test	0.069 ± 0.023	0.045 ± 0.007	0.059 ± 0.003	0.072 ± 0.004	0.101 ± 0.020
		[0.021–0.177]	[0.021–0.054]	[0.054–0.065]	[0.065–0.081]	[0.081–0.177]

**Table 2:** Comparison of the sum of absolute differences (SAD) between “Difference Image without VoxelMorph” and “Difference Image with VoxelMorph” (subgroups) (SAD average ± standard deviation).

	Breast Area (%)		Mammary Gland Content Ratio (%)		Compressed Breast Thickness (mm)		Difference Image without VoxelMorph (SAD)	
	Without	With	Without	With	Without	With	Without	With
Group 1	0.0540 ± 0.013	0.0402 ± 0.009	0.0791 ± 0.026	0.0621 ± 0.021	0.0452 ± 0.014	0.0381 ± 0.011	0.0563 ± 0.007	0.0438 ± 0.007
Group 2	0.0600 ± 0.014	0.0468 ± 0.009	0.0724 ± 0.026	0.0581 ± 0.020	0.0589 ± 0.022	0.0463 ± 0.018	0.0691 ± 0.003	0.0545 ± 0.007
Group 3	0.0727 ± 0.018	0.0576 ± 0.013	0.0658 ± 0.018	0.0511 ± 0.015	0.0723 ± 0.023	0.0566 ± 0.019	0.0724 ± 0.004	0.0579 ± 0.009
Group 4	0.0903 ± 0.026	0.0750 ± 0.019	0.0596 ± 0.017	0.0482 ± 0.015	0.1005 ± 0.025	0.0786 ± 0.019	0.0790 ± 0.020	0.0633 ± 0.018

## Data Availability

The data used in this study are available upon request from the corresponding authors.
